# Health care waste management among health workers and associated factors in primary health care facilities in Kampala City, Uganda: a cross-sectional study

**DOI:** 10.1186/s12889-019-6528-4

**Published:** 2019-02-18

**Authors:** Solomon Tsebeni Wafula, Julian Musiime, Frederick Oporia

**Affiliations:** 1 Department of Disease Control and Environmental Health, School of Public Health, Makerere University, P.o Box 7072, Kampala, Uganda; 2 International Health Sciences University (IHSU), Kampala, Uganda

**Keywords:** Healthcare waste, Waste management, Healthcare workers, Primary health care facilities, Kampala, Uganda

## Abstract

**Background:**

Healthcare wastes (HCWs) are one of the most hazardous wastes globally; second to only radiation waste. HCW management needs to be prioritized because of the devastating effects on human health and environment if not well managed. Health workers play a crucial role in management of HCWs. This study investigated the management of HCWs among health workers and associated factors in primary health care facilities in Kampala City, Uganda.

**Methods:**

We conducted a cross-sectional health facility survey in 8 primary health care facilities in Kampala City from March to April 2017. We interviewed health workers who provided data on socio-demographic characteristics, knowledge, attitudes and practices on HCW management. Prevalence ratios (PRs) and their corresponding 95% confidence intervals were used as a measure of association between HCW management and associated factors. The PRs were obtained using a multivariable modified Poisson regression using a generalized linear model of Poisson family with a logarithm as the canonical link function, with robust standard errors while applying a forward elimination method.

**Results:**

A total of 200 health workers responded to the survey; Knowledge of HCW management  was high 143 (71.5%, 95% CI (65.2–77.8)). About 160 (80.0%) wore appropriate personal protective wear when handling HCWs. Overall, 148 (74.0%, 95% CI (67.8–80.1) had satisfactory HCW management practices. Health workers with diploma education (Adjusted PR = 1.49, 95% CI (1.13–1.96), working in the teenage corner (Adjusted PR = 1.10, 95% CI (1.01–1.29), previous training on HCW management (Adjusted PR = 1.19, 95% CI (1.01–1.42) and those who thought HCW management was important (Adjusted PR = 2.81, 95% CI (1.22–6.47) were more likely to have satisfactory HCW management practices.

**Conclusion:**

The practices of health workers on HCW management were largely satisfactory. Higher odds of HCW management were determined among health workers with diploma education, previous HCW management trainings and among those who perceived HCW management as important. There is need to organize HCW management trainings in order to improve their HCW management practices among health workers.

## Background

Health care wastes are considered the second most hazardous wastes globally after radiation waste [1]. Healthcare wastes encompasses various forms of waste such as sharps, human body parts, blood, chemical wastes, pharmaceutical wastes, and medical devices [2]. These wastes are majorly generated from hospitals and primary care facilities, laboratories, mortuaries, autopsy centres, laboratories, blood banks, nursing homes among others [3]. Healthcare wastes can have devastating effects on human health if not properly handled [4]. Mismanagement of HCWs can result into various hospital-acquired infections, occupational health hazards and food contamination [5]. In addition, mismanaged wastes such as sharps contaminated with blood facilitates transmission of infections such as hepatitis B, hepatitis C, HIV/AIDs and other viral infections [2, 6, 7]. Health care workers, patients, workers in support services, visitors to health facilities, waste handlers, scavengers, fetuses in the wombs, and the general public are susceptible to these infections [2, 8].

In developing countries especially in Africa, healthcare waste has not received the much needed attention that it deserves [9–11]. This is because of the inadequate resources in these countries resulting into low priority for HCW management [12]. In many countries, there is limited segregation of hazardous and medical wastes and usually mixed with non-infectious waste [13, 14]. Inadequate knowledge and unsatisfactory management practices among the health care workers are major challenges in the management of HCWs [15]. Previous research indicate that HCW management may be affected by lack of formal training, lack of knowledge on HCW management, limited interest from hospital administration [16–18].

In Uganda, the HCW generated is averaged at 92 Kilograms (Kg) per day in hospital while primary health care facilities (Level  IV health centers, level III health Centre and level II Health Centre) generate about 42Kg, 25 kg and 20 kg respectively on daily basis [19]. Moreover, most of these primary health care facilities lack proper HCW management facilities [20, 21]. Despite the policy guidelines on injection and HCW management developed by the Ministry of Health, Uganda, there is sufficient evidence that HCWs including in Kampala health facilities is not properly handled [22, 23]. Although health care workers are routinely involved in managing HCW at their workstations, there is little published data on HCW management among health workers and more especially in primary health care facilities. Previous studies [16, 23, 24] on HCW management in East Africa were mainly conducted in high level health facilities (hospitals) and not primary health care facilities. These studies also delved more on HCW at facility management level and did not explore HCW management among health workers. There is paucity of studies conducted to assess the management of HCWs among health workers in primary health care facilities and factors that may influence it.

We therefore set out to understand HCW management practices among health workers in primary health care facilities in Kampala and the factors that influence those practices.

## Methods

### Study design, setting and study population

The study was cross sectional in design and employed quantitative techniques of data collection. It was conducted in Kampala, Uganda’s Capital City between March to April 2017. According to 2014 census results, Kampala has an estimated resident population of 1,507,080 people with an annual growth rate of 2.02% [25].

This study was conducted among health workers in 8 primary health care facilities in Kampala. In Uganda, the health care system is organised into a four-tier system (i.e., hospitals, health centres of levels IV, III and II). Hospitals are considered high level facilities while health centres; II, III and IV are lower level and are classified as primary health care facilities. These primary health care facilities offer basic services for example Health centre IIs offer out-patients consultations, health centre IIIs offer inpatient care and some laboratory services. In addition to services offered at lower levels, Health centre of level IV offer some caesarean operations and blood transfusion services. The main waste streams generated in these facilities included sharps, pathological wastes, infectious wastes and general wastes.

The study units were the primary health care facilities and the study participants were health workers. In this study, we defined a health worker as a person who works at health care centre and is engaged in all actions aimed primarily on enhancing health of patients. These health workers included nurses, midwives, medical officers, laboratory personnel, counsellors and social workers. All selected health workers who consented were interviewed. Those who were absent on leave or medically not well were excluded.

### Sample size and sampling procedure

The Kish Leslie formula for cross sectional studies was used to calculate sample size of 231 health workers [26].

$$ \mathrm{Sample}\ \mathrm{size},\left(\mathrm{N}\right)=\frac{{\mathrm{Z}\upalpha}^2\mathrm{pq}}{\upsigma^2} $$

We considered, the following assumptions: Z score, Zα = 1.96; prevalence, *p* = 16.3% (adopted from a study on adherence to waste disposal guidelines among health workers in Kenya) [27], a precision, σ = 5% and non-response rate of 10%. It is important to note that actual population of health workers in these primary health care facilities in Kampala was small (< 500).

Proportionate sampling was done to select a specific number of health workers from each of the 8 primary health care facilities based on the number of health workers in each health facility (Table 1). This was based on the numbers of health workers obtained from Kampala Capital City Authority (KCCA).

**Table 1:** Number of health workers planned for interview at each facility

	Health facility	Number of health workers (*n*)	Percentage (%)
1	Kisenyi Health centre	45	19.5
2	Bukoto Health Centre	12	5.2
3	Kawaala health centre	36	15.6
4	Kisswa health centre	40	17.3
5	Komamboga health centre	22	9.5
6	Kitebi health centre	25	10.8
7	Kisuggu health centre	41	17.7
8	City hall clinic	10	4.3
	TOTAL	231	100

From each of the health facilities, stratified proportionate sampling was again employed to select the different cadres of health workers (nurses, midwives, medical officers, laboratory personnel, counsellors and social workers).

### Data collection

We used a semi-structured questionnaire to collect quantitative data from health workers on socio-demographic characteristics, knowledge of HCW and perceptions of risk due to HCW and to capture health workers’ HCW management practices such as segregation, availability of necessary accessories, occupational safety and health in their work stations. The tools were developed based on findings from published studies [1, 9, 23]. We pretested data collection tools with 15 health workers in a health centre in the neighbouring Wakiso district. Pretesting was done to mitigate any errors and avoid ambiguity in preparation for actual data collection. This facility was chosen because it is close to Kampala and has similar HCW challenges as the primary health care facilities in Kampala.

### Data management and analysis

Data were checked daily for completeness, entered using EpiData version 3.02 (EpiData association, Denmark). We used Stata 14.0 (Statacorp Texas; USA) for cleaning and analysis. To determine the status of HCW management (Outcome variable), which was classified as either satisfactory or unsatisfactory, seven questions were asked on practices on healthcare waste management with the responses “Yes and No”. “No” was assigned 0 and “Yes” was assigned 1. We used mean scores to divide the HCW management status of health workers into two groups as suggested in the SAGE encyclopaedia of communication research methods [28]. As such, respondents who scored above 4.995 (average score) on 7 questions were considered as having satisfactory healthcare waste maintenance practices and those that scored less than 4.995 were deemed not to have satisfactory healthcare waste management practices. Prevalence rate ratios (PRs) were computed using a generalized linear model of the Poisson family with the logarithm as the canonical link function, with robust standard errors while applying a forward elimination method. This was done to measure the association between the outcome and independent variables. Prevalence rate ratios (PRs) were used instead of odds ratios since the prevalence of the outcome variable was > 10%, yet logistic regression’s odds ratios tend to overestimate the relative risk in such instances [29, 30]. Simple models consisting of the outcome and one independent variable were run to obtain the crude PRs. In the multivariable model, variables that were significant at simple models (*p* < 0.05) and those that had plausibility with the outcome were included. The crude and the adjusted PRs and their corresponding 95% confidence intervals were presented. Level of knowledge which was a predictor variable was also classified as either good knowledge or poor knowledge, five questions were asked on knowledge on healthcare waste management with the responses “Yes and No”. “No” was assigned 0 and “Yes” was assigned 1. Respondents who scored above 4.6 (average score) were considered as having a good knowledge and those that scored less than 4.6 were deemed to have poor knowledge on HCW management. Level of knowledge was however excluded in the final regression model due to collinearity. Strengthening the reporting of observational studies in epidemiology (STROBE) guidelines informed the reporting of our study [31].

## Results

### Socio-demographic characteristics of participants

A total of 200 health workers participated in the study representing response rate of 86.6%. Participation was skewed towards the female health workers since only 53(26.5%) were males. Two-thirds were married 135 (67.5%), aged 18–35 years 131 (67.2%). Most participants identified themselves as Christians 161 (80.5%), 151 (75.5%) as nurses or midwives by training and 136 (68.0%) held a diploma as their highest education level (Table 2).

**Table 2:** Socio-demographic characteristics of 200 health workers

Variables	Frequency (*n* = 200)	Percentage (%)
Total	200	100
Gender		
Female	147	73.5
Male	53	26.5
Age (Years)		
18–35	131	65.5
> 35	69	34.5
Marital status		
Married	135	67.5
Unmarried	65	32.5
Religion		
Christian	161	80.5
Non-Christian	39	19.5
Job designation		
Nurse/midwife	151	75.5
Medical officer	16	8.0
Counsellor	18	9.0
Others^a^	15	7.5
Department		
Outpatient department	124	62.0
Maternity ward	39	19.5
HIV section	10	5.0
Teenage Corner	10	5.0
Other departments	17	8.5
Level of education		
Diploma	136	68.0
Higher secondary (A’ level)	34	17.0
Lower secondary (O′ level)	30	15.0

^a^Other designations included laboratory personnel, social work

### Knowledge and attitudes of health workers on HCW management

Knowledge of health workers on HCW management was high 143 (71.5%). Majority knew the risks associated with HCWs 182(91.0%), the right colour codes for the bins for different wastes forms 179 (89.5%). About 120 health workers (60.0%) had been trained on HCW management and 41 (34.2%) had attended at least three trainings. As regards attitudes, most 181 (90.5%) indicated that HCW management was important and 186 (93.0%) agreed that strict implementation was necessary for proper HCW Management in health facility setting (Table 3).

**Table 3:** Knowledge and attitudes of Health workers on HCW management

Variables	Frequency (*n* = 200)	Percentage (%)
Knowledge on HCW Management		
HCW management is		
Activities and actions to manage waste from its inception to final	190	95.0
Collection of waste from one central place	03	1.5
Generation of waste	07	3.5
Knowledge of risks of poor HCW management		
Yes	182	91.0
No	18	9.0
Knowledge of the level of Risk to health workers		
High	182	91.0
Low	18	9.0
Infections due to poor HCW management can be avoided		
Yes	179	89.5
No	21	10.5
Ever attended training in HCW management		
Yes	120	60.0
No	80	40.0
Number of Trainings attended (*n* = 120)		
1–2	79	65.8
3 and above	41	34.2
Knowledge of bin colour codes for different waste streams (waste segregation)		
Know (Red, yellow, black)	179	89.5
Do not know	21	10.5
Knowledge scores (CIs for High Knowledge)	(65.2–77.8)	
Poor knowledge	57	28.5
High Knowledge	143	71.5
Attitudes on healthcare wastes management		
HCW management is important		
Yes	181	90.5
No	19	9.5
Strict implementation is necessary for proper HCW management		
Yes	186	93
No	14	7.0
HCW management is a serious problem		
Yes	182	91.0
No	18	9.0

*CIs*: Confidence Intervals

### Experiences and practices on HCW management

Overall, most 188 (94.0%) health workers had a waste bin around their working area and 144 (72.0%) wore appropriate personal protective equipment. Only 56 (28.0%) reported ever being affected by effects of poor HCW management with needle pricks 69.6% (39/56) and infections 26.8% (15/56) being the most reported effects / accidents. Nearly three quarters 148 (74.0%) were categorized as having satisfactory HCW management practices (Table 4).

**Table 4:** Practices of health workers on HCW management

Variables	Frequency (n = 200)	Percentage (%)
Presence of litter/waste bins in work station^b^		
Yes	188	94.0
No	12	6.0
Waste bins colour coded^2^		
Yes	164	82.0
No	36	18.0
Uses general pit to dispose general waste^b^		
Yes	71	35.5
No	129	64.5
Health workers dispose pathological wastes in the placenta pit ^b^		
Yes	163	81.5
No	37	18.5
Presence of Expired drugs at the work station^b^		
Yes	97	48.5
No	45	22.5
Did not know	58	29.0
Disposal of expired drugs		
Burning	18	9.0
Waste bin company	102	51.0
Return to national medical stores	76	38.0
Did not know	4	2.0
Use of Personal protective equipment (gloves, nose masks, clinical coats)^b^		
Yes	160	80.0
No	40	20.0
Regularity of use of Personal protective equipment^b^		
Always	144	72.0
Occasionally	56	28.0
Ever been affected due to poor HCW management		
Yes	56	28.0
No	144	72.0
Effects of poor HCW management (*n* = 56)		
Needle pricks	39	69.6
Infection	15	26.8
Spills of Blood	02	3.8
Ever reported injury to infection and control Unit		
Yes	35	17.5
No	165	82.5
Health care waste management status (CIs for satisfactory HCW management)	(67.8–80.1%)	
Satisfactory	148	74.0
Unsatisfactory	52	26.0

^b^Variables used in ascertaining HCW management status

### Independent predictors for HCW management among health workers

Compared to health workers with higher secondary education, health workers with a diploma were 1.49 times more likely to have satisfactory waste management practices (Adjusted PR = 1.49, 95%CI (1.13–1.96)), *p value* = 0.005). The odds of satisfactory waste management practices were 1.1 times higher among health workers in teenage corner as compared to those working in the outpatient clinic (Adjusted PR = 1.10, 95% CI (1.01–1.29)), *p value* 0.043). Participants who had been trained on HCW management were 1.19 times more likely to have satisfactory HCW management practices compared to those who had not received the training (Adjusted PR = 1.19, 95%CI (1.01–1.42)), *p value* = 0.042). Those who considered HCW management as important were 2.81 times more likely to observe satisfactory HCW management practices as compared to those who thought otherwise (Adjusted PR = 2.81, 95%CI (1.22–6.47)), *p value* = 0.016) (Table 5).

**Table 5:** Independent predictors of HCW management among health workers

Variables	Frequency (*n* = 200)	Proportion %	Crude PR 95% (CI)	Adjusted PR 95% CI	*P* value
Sex					
Male	53	26.5	1	1	
Female	147	73.5	1.04 (0.86–1.27)	1.17 (0.99–1.40)	0.068
Age (Years)					
18–35	131	65.5	1	1	
35 and Above	69	34.5	1.22 (1.05–1.43)*	1.13 (0.99–1.30)	0.060
Marital status					
Married	135	67.5	1		
Unmarried	65	32.5	0.99 (0.84–1.19)		
Religion					
Christian	161	80.5	1		
Non-Christian	39	19.5	0.84 (0.65–1.08)		
Job designation					
Nurse/midwife	151	75.5	1		
Medical officer	16	8.0	0.83 (0.56–1.22)		
Counsellor	18	9.0	0.88 (0.63–1.24)		
Others (laboratory personnel, social workers)	15	7.5	1.06 (0.81–1.38)		
Department					
Outpatient department	124	62.0	1	1	
Maternity ward	39	19.5	0.79 (0.61–1.04)	0.83 (0.66–1.03)	0.092
HIV section	10	5.0	0.52 (0.24–1.11)	0.55 (0.26–1.15)	0.111
Teenage Corner	10	5.0	1.29 (1.17–1.42)*	1.10 (1.01–1.29)*	0.043
Other departments	17	8.5	1.06 (0.84–1.35)	1.14 (0.89–1.47)	0.285
Level of education					
Higher secondary	34	17.0	1	1	
Diploma	136	68	1.69 (1.20–2.39)*	1.49 (1.13–1.96)*	0.005
Lower secondary	30	15.0	1.07 (0.66–1.72)	1.14 (0.77–1.68)	0.523
Knowledge of risks of poor HCW management					
Did not know	10	5.0	1		
Know	190	95.0	1.51 (0.80–2.82)		
Knowledge of risk of HCW to health workers					
High	182	91.0	1	1	
Low	18	9.0	1.73 (1.03–2.92)*	1.09 (0.67–1.79)	0.717
Ever attended training in HCW management					
No	80	40.0	1	1	
Yes	120	60.0	1.30 (1.08–1.58)*	1.19 (1.01–1.42)*	0.042
Knowledge of colour codes					
Did not know	21	10.5	1		
Know	179	89.5	1.12 (0.82–1.54)		
Overall Knowledge^c^					
Low	57	28.5	1		
High	143	71.5	3.77 (1.92–7.39)		
HCW management important					
No	19	9.5	1	1	
Yes	181	90.5	3.78 (1.57–9.07)*	2.81 (1.22–6.47)*	0.016
HCW management a serious problem					
Not serious	18	9.0	1		
Very serious	182	91.0	1.36 (0.89–2.08)		
Ever been affected by HCW					
No	144	72.0	1.		
Yes	56	28.0	0.99 (0.82–1.19)		
Ever reported injury to infection and control Unit					
No	165	82.5	1		
Yes	35	17.5	1.05 (0.86–1.29)		

*PR* Prevalence ratios, *CI* Confidence Interval, *p < 0.05

^c^Not included in multivariable model due to collinearity

## Discussion

This study aimed at understanding management of HCWs and associated factors among health workers in primary health care facilities in Kampala, Uganda. Our findings show that majority of the health workers had high knowledge on HCW management; knew how wastes are segregated and the risk to health. Most of them, used waste bins and wore personal protective wear when handling HCWs. A high proportion had satisfactory HCW management practices. Health workers in teenage corner, those who had attended training on HCW management and those who thought that HCW management was a challenge had higher odds of having satisfactory HCW management practices.

Concerning Knowledge on HCW management, most health workers had high knowledge especially on waste segregation and level of risk to health posed by HCWs. Similar findings have been reported in other studies where most health workers were aware of the risk of hazards such as injuries, infections (HIV/AIDS, Hepatitis B and C), and environmental pollution caused by improper HCW management [32, 33]. The prior trainings on HCW management that most health workers had, partly explains the high knowledge. In our study, 60% of participants had prior trainings consistent with what has been reported in other studies which indicated that most health workers had trained on HCW management. Training on HCW management is considered critical to success of any waste management programme; It improves knowledge of health workers, increases their cooperation with HCW programmes and also impacts on their practices on HCW management [34–36]. It is therefore important to intensify training for all health workers with emphasis on implications of proper HCW management on costs and risks to human and environmental health. Most health worker believed that strict enforcement is essential for proper HCW management. Many scholars have recommended strict enforcement; adding that this has to be complemented with continuous trainings [37, 38].

Wearing personal protective equipment (PPE) such as gloves, masks, clinical coats, shoes helps to minimise exposure to infections and injuries [39]. In this study, most health workers wore appropriate (PPE) which is a good practice since it minimises risk of contact with the waste. Our findings did not corroborate with findings of a cross sectional study conducted in a Tanzanian Municipality in which most health workers did not wear appropriate personal protective gear [40]. The low usage in the aforementioned study was attributed to the fact that health workers were not provided with protective gear by their employees. It is appropriate to ensure adequate provision of PPE and then supervision for proper and consistent use.

Generally, the practices of health workers on HCW management were satisfactory which relates to appreciable knowledge as there was significant association between practice and knowledge scores (*p* < 0001). The satisfactory practices suggest that health workers might be less likely to experience adverse effects associated with poor handling. Our finding corroborates with findings of a similar study conducted in Egypt in which most health workers had satisfactory practice scores [41]. It was found that health workers with diploma education were more likely to have satisfactory HCW management practices as compared to those with higher secondary education, a finding which is understandable given that a diploma generally offers better opportunity to acquire extensive knowledge on HCW management and yet such opportunity is almost non-existent in secondary school. Continued professional development may help improve the practices of health workers on management of HCWs.

Health workers who had received training on HCW management were more likely to have satisfactory practices. A possible explanation may be because they are able to put into practice what they have trained in. Our findings also support the findings of a similar study in Ethiopia which revealed that health workers trained on healthcare waste management were more likely to exhibit satisfactory practices on HCW management [42]. Trainings should therefore be intensified as they have shown to improve practices of health workers regarding how they handle HCW.

Health workers who thought that HCW management was important were more likely to have satisfactory HCW management practices as compared to those who thought otherwise. This finding is understandable because it is expected that such health workers based on their attitude would take practical measures to properly handle HCWs. It has been shown that health workers are more likely to be cautious and take necessary measures when they realise that HCW poses risk [42]. Health workers in the teenage corner were more likely to have satisfactory HCW management practices as compared to those in the outpatient clinic. This is understandable because further analysis found that all health workers in the teenage corner had diploma education unlike in the Outpatient department. The fact that higher education levels were associated with satisfactory practices may have modified the association between department and HCW management.

### Limitations

This study had some limitations; firstly, the sample size was relatively small which limits the generalizability of the results. The second limitation is that some variables that would have improved the assessment of Knowledge and attitudes even further were missed in the design of the tool. Future studies should consider other variables including those in the WHO checklists on medical waste management. Notwithstanding the limitations, the study provides useful information about management of HCWs among health workers and associated factors in primary health care facilities in Kampala, Uganda.

## Conclusions

Health workers in primary health care facilities in Kampala, Uganda generally have satisfactory practices on HCW management. Health workers with at least diploma education and those trained on HCW management were more likely to have satisfactory HCW management practices. Refresher trainings on HCW management and continuous supervision are therefore important in promoting proper HCW practices among health workers.

## Abbreviations

HCW: Health Care Waste
HIV: Human Immunodeficiency Virus
IHSU: International Health Sciences University
KCCA: Kampala Capital City Authority
PPE: Personal protective Equipment
PRs: Prevalence Ratios
WHO: World Health Organization
